# Dentists’ Satisfaction With the Dental Amalgam Phase Down Training Programme

**DOI:** 10.7759/cureus.90312

**Published:** 2025-08-17

**Authors:** Bernina K Kisumbi, Loice W Gathece, Olivia A Osiro, Susan W Maina

**Affiliations:** 1 Department of Dental Sciences, University of Nairobi Dental Hospital, Nairobi, KEN

**Keywords:** alternative restoratives, continuous professional development, dental amalgam, dentists, gender, kenya, minamata, phase-down, satisfaction, training

## Abstract

Background: Dental amalgam phase down (DAPD) has ushered in a paradigm shift towards the use of adhesive alternative restorative dental materials and dental caries prevention. The emergence of a new restorative practice model has created a gap among some dentists, highlighting the need to reorient their knowledge and skills.

Objectives: The objectives of this study were to assess dentists' level of satisfaction with a DAPD training programme and identify associations between sociodemographic characteristics and satisfaction levels, in Kenya, a low- and middle-income country.

Materials and methods: A three-hour online training programme was developed, and a sample of n=260 dentists was targeted. Data were collected on socio-demographic characteristics and from thirteen 5-Likert scale questions that evaluated participants’ satisfaction with the aims, usefulness, methodology, content and duration of the course. Data were analysed using descriptive statistics, with frequencies and proportions for categorical sociodemographic information and level of satisfaction scores, while chi-square and multivariate logistic regression were used to analyse the influence of sociodemographic information on the level of satisfaction with the statistical significance level set at p=0.05.

Results: A total of 216 (83.1%) dentists participated and completed the online questionnaire at the end of training, which represents 28.8% of the dentists whose contact information was retrievable as of 2022. Of these, 141 (65.3%) were men, while 130 (60.2%) were aged between 20 and 40 years. The majority obtained their primary degree in Kenya (190; 88.0%) and practised restorative dentistry (187; 86.6%). The dentists rated the training highly. The scores ranged from 3.74 to 4.63 with an average score of 4.43 on the 5-point Likert scale, indicating that they were highly satisfied with the DAPD training programme. Multivariate logistic regression showed that age, gender, year of qualification, county of workstation and practising restorative dentistry were positively associated with satisfaction. Dentists who practised in the cities were more than six times likely to be satisfied (aOR 6.1; CI: [1.3-8.554], p=0.012). Gender was statistically significant, such that male dentists were more satisfied (aOR 3.1; CI: [1.6-3.65], p=0.019).

Conclusion: The majority of the dentists were satisfied with the content of the training modules, mode of delivery and its usefulness in phasing down the use of dental amalgam. The strongest association was observed between practising restorative dentistry and satisfaction. Longitudinal training approaches to evaluate how satisfaction with training translates into changes in clinical practice would provide insight.

## Introduction

Training programmes play a key role in navigating the dynamic and challenging oral health care delivery setting in order to realise the requisite changes in practice [1]. The implementation of dental amalgam phase down (DAPD) as recommended by the Minamata Convention on Mercury mirrors patients' demand for tooth coloured restorations, thus gradually eclipsing the use of dental amalgam in posterior teeth worldwide. Whereas older dentists possess mastery of the principles and techniques of the use of dental amalgam, some may lack capacity to transition to the application of quality posterior dental amalgam alternative restoratives (DAARs) and the emerging alternative paradigms such as minimal intervention dentistry (MID) [2-4]. In particular, some dentists have highlighted their need to be trained on the appropriate use of alternatives [5,6], including the newer versions such as bulkfill dental resin composites, alkasites and glass hybrids [2,5,7].

The most widely used mode of training is the continuous professional development (CPD) model, and some studies have reported high levels of satisfaction with these courses among dentists [8-11]. However, there is a paucity of such studies on satisfaction with training among dentists, including on DAPD. In Norway, at the start of phasing down the use of dental amalgam, a day-long CPD was used to retrain dentists on the use of dental resin composites in posterior teeth, the most commonly used DAAR [12]. Though no satisfaction rating was done immediately after the training, one and a half decades later and a year after the phasing out of dental amalgam, dentists were found to be satisfied with the use of posterior resin composites and MID [12,13]. Moreover, studies have reported high levels of dentists’ satisfaction with training through various CPD modes, including face-to-face [11,14], online [8,15], and e-learning [10,16]. One of the methods of assessing satisfaction with training is the use of participants’ self-rating on a perception scale, and this has been employed in several studies [17-20]. Percentage frequencies of participants have also been utilised to capture data on satisfaction with training courses [11,17]. Appraising the various aspects of the training, such as participation, satisfaction and learning, can serve as key criteria for evaluating a programme [21]. Nevertheless, the hallmark of evaluating health practice CPDs is the commitment to change and subsequent intended practice and patient outcomes. The majority of studies evaluating CPD courses do so through satisfaction surveys [22], and training effectiveness models have been employed to capture data on the reaction to the training. In Kirkpatrick’s four-level model, satisfaction is evaluated in level I using Likert scale questions, and it has been deemed easy to report [18]. Alternatively, in the Moore seven-level model, satisfaction is evaluated in levels 1 and 2, and it is well-known in continuous medical education [23]. The DAPD is a complex agenda that will translate to a fundamental change in the way dentists have practised for years [24,25]. Dentists are key stakeholders in the Minamata Convention on mercury and the DAPD; thus, a training programme provides an avenue to build their capacity for change in clinical practice and participation in policy development [3]. Moreover, satisfaction with training has been reported to impact readiness to transition through normative commitment to the intended objectives. Hence, the aim was to assess dentists' level of satisfaction with a DAPD training programme and identify associations between sociodemographic characteristics and satisfaction levels, in Kenya, a low and medium-income country. 

## Materials and methods

A three-hour online DAPD training programme was designed and developed for implementation over two evenings among a sample of n=260 dentists. The sampling technique, sampling frame and training curriculum are outlined in the study by Kisumbi et al. [26]. Dentists who neither performed restorative procedures nor planned to do so after the training were excluded. Anonymous online questionnaires were piloted among 26 dentists and used to collect data on participants’ socio-demographic information, gender, age, qualification, country of training, sector of practice and practising restorative dentistry. Thirteen questions, developed in line with Kirkpatrick's model, evaluated participants’ satisfaction with the aims, usefulness, methodology, content, and duration of the course (Appendix). Data collection was conducted at a single point in time, immediately after the final training session. The participants’ reaction to the DAPD training programme was evaluated, corresponding to Kirkpatrick’s level I (reaction) immediately after the last training session. The dentists scored their level of satisfaction on a scale of (1-5), with 1 representing “strongly disagree” and 5 representing “strongly agree”. Results were considered satisfactory for the questions when scored 4 or 5. Data were analysed using descriptive statistics, including frequencies and proportions for categorical sociodemographic information and level of satisfaction scores. Chi-square and multivariate logistic regression were used to analyse the influence of sociodemographic information on the level of satisfaction, with the statistical significance level set at p=0.05.

## Results

A total of 216 (83.1%) dentists attended the training and completed the questionnaire, which represents 28.8% of the dentists whose contact information was retrievable as of 2022. Of these, 141 (65.3%) were men, while 130 (60.2%) were aged between 20 and 40 years. A majority obtained their primary degree in Kenya (190; 88.0%) and practised restorative dentistry (187; 86.6%). Only a minority worked in the public sector and rural counties (68; 31.4%). Overall, the participants rated the training highly (above 3.74 on the 5-point Likert scale), and almost all 199 (92.1%) were satisfied with the training programme. The item to which most participants 158 (73.1%) strongly agreed with was " The topics covered were suitable for the use of alternatives in phasing down the use of dental amalgam", whereas the item with the highest percentage of participants who strongly disagreed was " Overall I was satisfied with the course" (7; 3.2%) (Table 1). 

**Table 1:** Frequency distribution of satisfaction of the study participants with the DAPD training programme (n= 216) Data is presented as frequency (n), %, Mean ± Standard deviation (SD); MID: Minimally intervention dentistry; DAPD: Dental amalgam phase down; DAARs: Dental amalgam alternative restoratives

Variable	Strongly Disagree %	Disagree %	Neutral %	Agree %	Strongly Agree %	Mean ± SD
Overall, I was satisfied with the course	7 (3.2)	5 (2.3)	5 (2.3)	61 (28.2)	138 (63.9)	4.47±1.77
The aims of the training were clearly outlined	2 (0.9)	6 (2.8)	5 (2.3)	70 (32.4)	133 (61.6)	4.51±1.84
The aims of the training were met	2 (0.9)	2 (0.9)	7 (3.2)	78 (36.1)	127 (58.8)	4.51±1.45
The training was well organised	2 (0.9)	2 (0.9)	62 (28.7)	150 (69.4)	3 (1.5)	3.74±0.92
The duration of the course was adequate	2 (0.9)	6 (2.8)	59 (27.3)	13 (6.0)	137 (68.8)	4.40±1.08
The registration for the training was easy	3 (1.4)	12 (5.6)	22 (10.2)	66 (30.6)	113 (52.3)	4.27±1.05
Logging into the training sessions was unproblematic	1 (0.5)	1 (0.5)	8 (3.7)	97 (44.9)	109 (50.5	4.44±1.02
The online mode of delivery of the training was appropriate	2 (1.0)	1 (0.5)	4 (1.9)	72 (33.3)	137 (63.4)	4.58±1.27
The content on MID was applicable in DAPD	2 (0.9)	1 (0.5)	7 (3.2)	90 (41.7)	116 (53.7)	4.47±1.44
The content of the lecture on DAARs was helpful	1 ( 0.5)	2 (0.9)	14 (6.5)	68 (31.5)	131 (60.6)	4.51±1.24
The knowledge covered in lecture 1 on DAPD was adequate	2 (0.9)	12 (5.6)	10 (4.6)	84 (38.9)	108 (50.1)	4.52±1.35
The topics covered were suitable for use of DAARs in phasing down the use of dental amalgam	2 (0.9)	2 (0.9)	12 (5.6)	42 (19.5)	158 (73.1)	4.63±1.36
The training was beneficial to restorative dental practice	3 (1.4)	2 (0.9)	7 (3.2)	74 (34.3)	130 (60.2)	4.51±1.39

Bivariate analysis to evaluate the relationship between sociodemographic characteristics and satisfaction revealed that five out of the eight sociodemographic variables were statistically significantly associated with satisfaction (p <0.05). Among these, being male and practising restorative dentistry had a statistically highly significant association with satisfaction (p = 0.001) (Table 2).

**Table 2:** Bivariate analysis of the influence of sociodemographic characteristics on satisfaction of the dentists Data is presented as frequency (n), % and p-value <0.05 is statistically significant *The four cities of Kenya

Variable		Satisfied (%)	Not satisfied (%)	Total (%)	Chi-square	p-value
Gender						
i.	Male	152 (93.8%)	10 (6.2%)	162(100.0%)	104.00	0.001
ii.	Female	47 (87.0%)	7 (13.0%)	54 (100.0%)		
Age in years						
i.	24 – 39	89 (92.7%)	7 (7.3%)	96 (100.0%)	37.46	0.010
ii.	40 – 50	91 (94.8%)	5 (5.2%	96(100.0%)		
iii.	>50	19 (79.2%)	5 (20.8%)	24(100.0%)		
Highest qualification						
i.	BDS	156 (96.9%)	5(3.1%)	161(100%)	58.73	0.621
ii.	Masters and above	43 (78.2%)	12(20.8%)	55(100%)		
Year of primary qualification						
i.	<1960	10 (71.4%)	4 (28.6%)	14 (100%)	48.34	0.004
ii.	1960 – 1979	37 (80%)	6 (20%)	43(100%)		
iii.	1980 – 1999	54 (94.7%)	3 (5.3%)	57(100%)		
	>1999	98 (96.1%)	4 (%)	102(100%)		
Country of obtaining basic dental degree						
i	Kenya	183(96.3%)	7 (3.7%)	190 (100%)	78.12	0.612
ii.	Other countries	16 (69.2%)	10 (30.8%)	26 (100%)		
Sector of practice						
i.	Public	43 (76.8%)	13 (23.2%)	56(100%)	36.41	0.476
ii.	Private	85 (75.2%)	28 (24.8%)	113(100%)		
iii.	Both public and private and faith based	35 (74.5%)	12 (25.5%)	47(100%)		
County of work station						
i.	Cities^*^	100 (94.3%)	6 (5.7%)	106 (100%)	36.98	0.002
ii.	Rural	99 (93%)	11(7%)	110 (100%)		
Practice restorative dentistry						
i.	Yes	120 (96%)	5 (4%)	125 (100%)	76.11	0.001
ii.	No	79 (86.8%)	12 (13.2%)	91(100%)		

A multivariate analysis of sociodemographic factors associated with satisfaction was conducted. The bivariate analysis showed statistically significant differences with age, gender, year of primary qualification, county of work station and practising restorative dentistry. On the other hand, sociodemographic variables, highest qualification, country of obtaining primary dental degree and sector of practice did not show any statistical significance in this study. Sociodemographic factors that showed statistically significant associations in the bivariate analysis were confirmed through multivariate analysis to account for potential confounding effects. Dentists who worked in cities were six times more likely to be satisfied with the DAPD training programme (aOR: 6.1; CI: [1.3- 8.554], p=0.012), Table 3.

**Table 3:** Multivariate logistics regression and satisfaction of the dentists Data is presented as frequency (n), % and p-value <0.05 is statistically significant. aOR: Adjusted Odds Ratio; CI: Confidence interval; RC: Reference category

Variable	Satisfaction (%)	aOR (95% CI)	p-value
Gender			
Female	162 (75.0%)	3.1(2.6-3.65)	RC
Male	54 (25.0%)		0.019
Age in years			
24 – 39	96 (44.7%)	3.1(1.6-4.7)	0.024
40 – 50	96 (44.4%)		RC
>50	24 (11.2%)		RC
Year of primary qualification			
<1960	14 (6.5%)	4.7(1.3-4.86)	RC
1960 – 1979	43 (19.9%)		RC
1980 – 1999	57 (26.4%)		0.004
>1999	102 (47.2%)		RC
County of origin			
City	106 (49.1%)	6.1(1.3-8.554)	0.012
Rural	110 (50.9%)		RC
Practice restorative dentistry			
Yes	125 (57.9%)	3.4 (2.0-5.73)	0.003
No	91 (42.1%)		RC

## Discussion

Dental practice has continually evolved in response to novel research inventions, new dental materials, technological advances and global trends in other sectors that influence the population and environmental health. In order to keep pace with this, training has predominantly been delivered through CPD models worldwide [8,10,11,16]. Nevertheless, there is a paucity of studies on satisfaction with training among dentists. To the best of our knowledge, this is the first study evaluating the level of satisfaction with a DAPD training programme, slightly more than a decade since the inception of the Minamata Convention on Mercury. The response rate in the current study was high, and the participants rated the DAPD training programme very highly, indicating a high level of satisfaction. The study employed the training model of CPDs, which is universally recognised [27] and widely utilised as a means of introducing new knowledge, skills and competencies among professionals, dentists included [28,29]. This may explain the high rating of the training programme. Further, the fact that the study was based on a relevant topical subject in global dental practice may have matched the Kenyan dentists' training needs at the time of the study [24]. Nevertheless, it cannot be ruled out that some element of social desirability bias may have influenced the satisfaction responses.

There is a paucity of literature on the influence of sociodemographic characteristics on satisfaction with training among dentists. Nevertheless, levels of satisfaction have been reported [8,11,14]. In the current study, the association of the five variables with satisfaction may be explained by correlations among some of the variables, age and year of primary qualification. Additionally, practising restorative dentistry and the type of workstation may also be related, as in Kenya, most restorative work is performed in comparatively well-equipped clinics that are predominantly located in urban areas. The results of the current study are similar to findings among paediatric residents, where male residents were more satisfied than female residents [30]. It can be speculated that in the present study, this may be due to the comparatively higher population of male dentists in Kenya [31]. However, the results differ from those of a study among dental students where there was no significant association with gender [19]. The present study reports a minority of dentists working in the public sector and in rural Kenya, which aligns with the current geographical distribution, but may also have been influenced by internet penetration. Therefore, the current satisfaction outcome cannot be extrapolated to the entire nation. The rating of the training programme in this study is slightly lower but similar to that of an online radiology study done among Taiwanese dentists, where satisfaction was higher with an average score of 4.77 out of a 5-point Likert scale, with 113 (96.6%) stating they were satisfied with the course [8]. In a study on an interactive learning module for online education to increase the forensic relevance of oral health records, participants indicated satisfaction; however, the rate was not reported [9]. Additionally, Safi et al. designed, implemented and evaluated a CPD course among Iranian dentists on non-clinical competencies that are essential for successful practice [11]. A slightly lower satisfaction level compared to the current study was reported; that is, 32 (70%) of the participants were satisfied and completely satisfied with the course, while in the current study, it was 199 (92.1%). The difference may be attributed to the fact that in the Iranian study, the CPD course was delivered face-to-face and was also the first of its kind among dentists, whereas in the present study, the online CPD delivery of the training programme may have presented easier access for dentists. The findings of this study align with those of an e-learning regenerative endodontics skill course by Kolcu et al., among dentists in Turkey who were highly satisfied with the course [10]. This reinforces previous studies that have reported dentists' satisfaction with online-based CPDs. Akin to the current study, oncology health professionals were highly satisfied with a modular blended learning course [20]. Apart from the high level of satisfaction with the training programme in this study, dentists also registered high levels of satisfaction with the duration of the programme. This may be attributed to the incorporation of a short study duration, which aligned with preferences identified in the pilot study. Additionally, the topic of the current study may have resonated with the training needs of Kenyan dentists at the time. This study's findings are consistent with findings of a previous study in which, following a short training on capacity building of dental faculty on the quality of multiple choice questions, high levels of satisfaction were reported [14]. Further, the study evaluated participants’ satisfaction using Kirkpatrick’s level I, akin to the present study. 

## Conclusions

Overall, the participants rated the DAPD training programme highly and agreed that its aims were effectively met. The majority of the dentists were satisfied with the topics covered on DAARs, the online mode of delivery and its usefulness in phasing down the use of dental amalgam. A trend of association of satisfaction with the training was observed between participants working in the cities and practising restorative dentistry, whereas the weakest association was found with age and gender. The satisfaction with the DAPD training programme is a positive reflection of the DAPD and the implementation of the recommendations of the Minamata Convention on Mercury agenda among dentists in Kenya. Longitudinal training approaches to evaluate how satisfaction with training translates into changes in clinical practice would provide insight. A key limitation of this study design is the absence of open-ended questions, which would have allowed participants to provide feedback on real-time concerns. Additionally, there is sampling bias, with more urban dentists participating compared to those working in rural counties.

## Appendices

**Table 4:** Questions for sociodemographic characteristics and the rating of the satisfaction level SD: Strongly disagree (1) D: Disagree (2) N: Neutral (3) A: Agree (4) SA: Strongly agree (5)

Variable	Responses				
Please indicate your gender	Male	Female			
Please indicate your current age	Number of years				
What is your highest qualification?	BDS	Master’s degree	PhD	Other please specify	
In which country did you study your basic dental degree?	Kenya	Other please specify			
Year of primary dental qualification?					
Which sector do you work in?	Public	Private	Both public and private	Faith based	Other please specify
In which county is your work station located?					
Do you practice restorative dentistry?	Yes	No			

No.	Item	SD	DS	NT	A	SA
	Overall, I was satisfied with the course					
	The aims of the training were clearly outlined					
	The training was well organised					
	The duration of the course was adequate					
	Logging into the training sessions was unproblematic					
	The online mode of delivery of the training was appropriate					
	The content on minimally invasive dentistry was applicable in phase down of dental amalgam					
	The content of the lecture on dental amalgam alternatives was helpful					
	The knowledge covered in lecture on dental amalgam phase down was adequate					
	The topics covered were suitable for use of alternatives in phasing down the use of dental					
	The training was beneficial to restorative dental practice					
	The registration for the training was easy					
	The aims of the training were met					
